# Emerging Technologies to Study the Glomerular Filtration Barrier

**DOI:** 10.3389/fmed.2021.772883

**Published:** 2021-11-25

**Authors:** Emma Gong, Laura Perin, Stefano Da Sacco, Sargis Sedrakyan

**Affiliations:** ^1^Division of Urology, GOFARR Laboratory for Organ Regenerative Research and Cell Therapeutics, Children's Hospital Los Angeles, Saban Research Institute, Los Angeles, CA, United States; ^2^Keck School of Medicine, University of Southern California, Los Angeles, CA, United States

**Keywords:** Proteinuria, kidney disease, single cell transcriptomics, spatial transcriptomics, kidney organoids, kidney-on-a-chip, glomerular filtration barrier

## Abstract

Kidney disease is characterized by loss of glomerular function with clinical manifestation of proteinuria. Identifying the cellular and molecular changes that lead to loss of protein in the urine is challenging due to the complexity of the filtration barrier, constituted by podocytes, glomerular endothelial cells, and glomerular basement membrane. In this review, we will discuss how technologies like single cell RNA sequencing and bioinformatics-based spatial transcriptomics, as well as *in vitro* systems like kidney organoids and the glomerulus-on-a-chip, have contributed to our understanding of glomerular pathophysiology. Knowledge gained from these studies will contribute toward the development of personalized therapeutic approaches for patients affected by proteinuric diseases.

## Background

The kidney performs filtration function of removing waste products and maintaining fluid balance in the body. The structural unit responsible for these processes in the kidney is the nephron constituted by the glomerulus and tubules (1). The filtration process occurs in the glomerulus, within the glomerular filtration barrier comprised of fenestrated glomerular endothelial cells, glomerular basement membrane [300–350 nm thick membrane containing type IV collagen, proteoglycans, laminin and nidogen (2)] and visceral epithelial cells (podocytes) (3). Parietal epithelial cells make up the structure of the Bowman's capsule. The ultrafiltrate that leaves the glomerulus passes through the Bowman's space on its way to entering the tubules where reabsorption and secretion processes lead to the production of urine. Proteinuria is defined as the presence of abnormal or increased amount of protein in the urine (such as neutrophil gelatinase-associated lipocalin (NGAL), kidney injury molecule-1 (KIM-1), cystatin C, α1-microglobulin and Tamm-Horsfall proteins (4). Proteinuria has been recognized as a marker of kidney disease since Hippocrates in 400 B.C. (5, 6) and is currently considered a key diagnostic indicator of renal progression. Impairment and/or dysfunction of different nephron compartments, glomeruli and tubules, have been shown to strongly contribute to initiation of proteinuria (7). Inversely, recent studies have also confirmed that high levels of proteinuria can directly damage the kidney (8). Thus, understanding the diverse functional roles of the cells comprising the glomerular filtration apparatus and the tubular structure in contributing to the development of proteinuria is critically important to finding new avenues or pathways to inhibit these processes. Different *in vitro* and *in vivo* studies have been conducted both in humans as well as in animal models, leading to important advances in our understanding of physiological changes causing proteinuria (4, 7–9). However, the ability to perform mechanistic studies in patients is not practical, and insights gained from animal studies are limiting due to differences between human and animal physiology. In this regard, emergent technologies, such as single cell and spatial transcriptomic as well as kidney organoids and microfluidic systems can greatly contribute to more in-depth understanding of the molecular origins of proteinuria, thus helping to elucidate the key molecular pathways responsible for the initiation and progression of these processes. Knowledge gained from these studies will help the identification of possible novel targets for therapeutic treatments for patients with chronic kidney disease. In this review we will describe these approaches, discuss their contribution to the field and pinpoint advantages and pitfalls.

## Single Cell Transcriptomic Applications to Study Kidney Disease and Proteinuria

Bulk RNA-sequencing (RNA-seq) approaches have been instrumental in elucidating important characteristics about kidney pathophysiology, but it was the development of the single cell RNA-seq (scRNA-seq) that greatly expanded our knowledge on kidney cell heterogeneity, allowing the characterization of renal cell types based on gene expression of few cells. This also opened new avenues for the comparison of transcriptomic profiles of normal renal vs. diseased renal tissues, creating new opportunities to uncover early mechanisms leading to proteinuria and kidney damage.

Several reports provide in detail characterization of renal and/or glomerular specific cell types from mice and humans using the scRNA-seq approach. Through unbiased clustering of scRNA-seq data Chung et al. successfully identified different cell types in the C57BL/6J mouse glomerulus, with podocytes, glomerular endothelial cells, and mesangial cells comprising greater than 90% of the cells, indicating the highly successful isolation of glomeruli (10). The remaining 10% of cells were identified as vascular smooth muscle cells, immune cells, parietal epithelial cells, and proximal tubular (PT) cells. Novel marker genes and gene signatures for mesangial cells (*Plvap, Prkca, Art3*, and *Nt5e*), vascular smooth muscle cells of the afferent and efferent arterioles (*Cnn1, Cygb)*, and new population of endothelial cells (*Dlk1, Ednrb*) were identified. To find a genetic and cell specific link to proteinuria, Park et al. analyzed the expression pattern of 29 human gene homologs in mice, which have been associated with monogenic inheritance of proteinuria in humans (11). Unlike earlier studies that implicated defects in endothelial cells and PT in the development of proteinuria (12–16), they concluded that podocyte dysfunction is the primary reason for proteinuria and could be the focus for targeted therapeutics based on the observations that 21 out of the 29 genes were found in only one cell type – the podocyte (11).

scRNA-seq has also been instrumental for investigating the transcriptomic changes in cell-type specific responses to injury within the glomerulus. Analysis of three different glomerular injury models (BTBR ob/ob mice, doxorubicin treatment, CD2AP-deficient mice) by Chung et al. showed minimal overlap in terms of transcriptional responses and the cell types involved, with the exception for mesangial cells, which showed persistent induction of genes involved in wound healing, including distinct patterns of expression for matrix (*Col4a1, Col6a3, Col8a1*) and chemokine (*Ccl2, Cxcl1, Cxcl13*) associated genes in each model (10). In contrast, in a glomerular nephritis mouse model, injection of nephrotoxic serum induced cytoskeletal regulation, cell adhesion and inflammatory response in podocytes on day 1 (peak proteinuria) that largely normalized on day 5, when proteinuria was nearly resolved. In podocytes, the response to injury was shown to be mediated through the Hippo pathway with upregulation of *YAP* and *TAZ* genes, and their known targets, including *Ctgf, Cyr61*, and *Axl*. The effects of injury were more prominent in TAZ (Wwtr1-/-) or YAP (Yap1-/-) knock-out mice at day 1 and exhibited delayed resolution, indicating that Hippo pathway proteins are essential for podocyte recovery after immune injury. Identifying the genes and pathways involved in the cell's programmed injury response holds potential to finding new biomarkers of kidney injury and proteinuria. In addition, scRNA-seq has been applied to study the role of sex differences on kidney function in health as well as during disease progression. In this regard, important differences in the segment 3 (S3) of the PT were identified. It was found that males had high expression of the *Cyp7b1* transcript while females had high expression of the *Slc7c12* transcript (17). The differential expression of genes in male PT cells could possibly be linked to their increased susceptibility to ischemia compared to their female counterparts. While no clear evidence has been found yet to corroborate these mechanisms in humans, if these findings are confirmed they can inform about novel sex-specific targets for the treatment of proteinuria.

In human, applications of scRNA-seq have been widely used to study different etiologies of CKD, including IgA nephropathy (18), diabetic nephropathy (19), lupus nephritis (20, 21) and others (22). Studies in IgA nephropathy revealed increased mesangial expression of novel genes, including *MATAL1, GADD45B, SOX4*, and *EDIL3*, and upregulation of genes enriched in inflammatory pathways including *TN, IL17*, and *NOD-like receptor* signaling in the tubules (18). In early diabetic nephropathy single nucleus RNA-seq showed increased immune cell activation and revealed cell type specific transcriptional signature consistent with increased potassium secretion, driven by alterations in *Na*+*/K*+*-ATPase, WNK1, NEDD4L*, and mineralocorticoid receptor expression, and decreased paracellular calcium and magnesium reabsorption (19). In lupus nephritis kidneys, 21 disease specific gene subsets specific for leukocytes, including myeloid, natural killer (NK), T and B cells were identified (21). Independently, using scRNA-seq, Chen et al. analyzed kidney biopsies affected by IgA nephropathy, lupus nephritis and membranous nephropathy and showed that podocytes from all these etiologies had increased expression of *FXYD5, CD74, B2M* (beta-2 microglobulin, a component of MHC class I molecules) when compared with a healthy donor (20). Increased *CD74* (a trafficking regulator and cell membrane receptor for macrophage migration inhibitory factor) mRNA expression has been associated with increased interaction between podocytes and immune cells in glomerulonephritis. In addition, *B2M* has pro-inflammatory properties (20). Since immune infiltration has been linked to proteinuria, further research into how the upregulation of CD74 expression may contribute to poor filtration would aid in our understanding of proteinuria development. Increased immune cell infiltration in the glomerulus in lupus nephritis has been shown to impair filtration and lead to proteinuria (23, 24) suggesting that these kind of studies could be pivotal for understanding the signaling cascades involved in the development of proteinuria.

While great advancements have been achieved with the use of scRNA-seq technology in the study of kidneys and acute and chronic renal diseases, there are technical limitations, ranging from depth of the sequencing and higher background noise that limits the ability to detect lowly expressed genes to high data variability that prevent more robust and in detail data analysis. There are also biological limitations that prevent comprehensive detection, analysis, and interpretation of scRNA-seq data. One such limitation is the so called “transcript dropout phenomenon”, defined by loss of transcription in large fractions of cells that usually occurs for low or moderately expressed transcripts. This may be explained by temporal fluctuations in the transcription, when the expression of protein is there, but its transcript may not be detectable for a large fraction of the time following initial higher transcriptional activity [a phenomenon called transcriptional bursting (25)]. There are also limitations regarding differentiating different renal cell types, like for example glomerular mesangial cells from other stromal mesenchymal cell types. Since the early focus of much research on the glomerular mesangial cells in the 1970's, the lack of molecular definition and absence of genetic tools have been major hurdles to study their functional role in glomerular injury. This is because many genes typically expressed by mesangial cells, such as *PDGFRA, PDGFRB, DES, GATA3* and *ITGA8* are also highly expressed in other stromal cells (10, 26). These studies unraveled that mesangial cells do not only possess a pericyte-like signature, but also a prominent fibroblast-like and vascular smooth muscle cell-like profile, suggesting that they represent a hybrid of pericyte-vSMC (vascular smooth muscle cells) and fibroblast.

In addition, the availability and use of different scRNA-seq platforms and library preparation methodologies [such as CEL-seq2/C1, Drop-seq, MARS-seq, SCRB-seq, Smart-seq/C1 and Smart-seq2 (27)] makes it difficult to make valuable comparisons between different studies (28). Much needed improvements in technical and computational approaches that constitute the basis of scRNA-seq will in the near future promote our ability to take full advantage of single cell analysis and enable the discovery of subpopulations of interest, benefitting our understanding of kidney health and disease.

## Spatial Transcriptomic Applications to Study Kidney Disease and Proteinuria

Spatial transcriptomics (ST) is an emerging new technology that enables the study of gene expression profiles in tissue histology sections while retaining the morphological information of the transcripts and their originating cell types (29). ST integrates quantitative transcriptomics (30–32) with high-resolution tissue imaging, spatially resolved *in situ* hybridization technologies (33–35) and unbiased bioinformatics analysis to facilitate the molecular characterization of tissue and cell structures. This is accomplished using barcode-based approaches (29, 36, 37), which in contrast to tissue dissection followed by sequencing-based transcriptomic profiling and fluorescence *in situ* hybridization (FISH) approaches, allows for whole transcriptome spatial profiling.

The morphological context of gene expression is of critical importance when studying cell-to-cell interactions or signaling between different segments of a functional tissue to understand the molecular mechanisms of tissue homeostasis vs. pathological developments. The advent of ST creates tremendous opportunity for understanding proteinuric kidney disease. By retaining the positional information of cells and individual nephrons, ST enables the study of disease heterogeneity one nephron or nephron compartment at a time, which is critically essential for understanding how transcriptional diversity contributes to mosaicism of tissue phenotypes which ultimately progresses to the development of proteinuria and renal failure.

Over the last decade, the utility of ST in biological research has grown substantially. Many studies on different mouse models of human disease as well as human specimen have been performed, but mainly in the cancer field (38–40). Several powerful commercial platforms have since emerged, including the Visium by 10X Genomics (US), the NanoString Technologies (US), Akoya Biosciences (US), Fluidigm, Canopy Biosciences (a Bruker company), Lunaphore Technologies (Switzerland), Vizgen (US) and RareCyte (US) that offer whole genome as well as custom ST solutions (41). To this date, very few studies reported the use of ST in the kidney setting, mainly in the acute kidney injury (AKI) setting (42–44).

Different regions of the nephron, due to their specific functional and metabolic needs have different susceptibility to AKI, and are, therefore affected variably (42). ST represents a powerful tool that can contribute to our understanding of the spatial orientation and cellular interactions in AKI providing nephron-segment-specific characterization of the disease heterogeneity. Applying the 10x Genomics Visium ST platform, Melo Ferreira et al. (43) interrogated epithelial cell-immune crosstalk in two murine AKI models, ischemia/reperfusion injury (IRI) and cecal ligation puncture (CLP). The distribution of *Havcr1* (hepatitis A virus cellular receptor 1, that produces the kidney injury molecule-1 protein) expression was found to be localized to the outer stripe region of the medulla in the IRI model and remained unregulated in the LCP model. The outer stripe in the IRI model co-localized with the expression of *Atf3* (activating transcription factor 3, a regulator of neutrophil migration) in the PT S3 cells. Importantly, 52.2% of Ly6G^+^ and CD11b^hi^ neutrophils in the IRI model were found localized in the outer stripe by immunofluorescence imaging. In the CLP model, 80.2% of the Ly6G^−^ and CD11b^hi^ infiltrating macrophages co-localized with cortical PT epithelial regions, while the NK cells were found in the cortex and outer stripe of the kidney in the ST data, suggesting that a subpopulation of PT cells with Atf3 expression may be responsible for neutrophils chemotaxis (43). A link between neutrophil recruitment and development of proteinuria has been suggested and is currently under intense investigation (44–46). These studies were done using human cells and mouse models.

In addition, areas of low-expression regions were identified when compared with sham control. In IRI, the low-expression regions had upregulated genes highly enriched in pathways associated with metabolism of amino acids and fatty acids, and injury response mechanisms including apoptosis, oxidative stress and the p38 MAPK cascade. Neutrophil migration and IL-17 signaling was associated with potential inflammatory response of neutrophils in the IRI model. In the CLP model in contrast, pathways enriched in low-expressing regions were associated with p53 signaling, cell cycle arrest, apoptosis, TNF signaling and macrophage differentiation (43).

In a mouse model of endotoxemia associated renal injury, Janosevic et al. used integration of Visium Spatial Gene Expression with scRNA-seq to map transcriptomic changes from early injury into the recovery phase of the disease (47). Using this method, spatial resolution of unique subclusters of endothelial cell populations were plotted. Subtypes of S3 PT called S3-Type 2 (S3T2) with unknown position in the nephron and characterized by expression of angiotensinogen (*Agt*) and other unique identifiers such as *Rnf24, Slc22a7*, and *Slc22a13* were localized to the outer stripe of the outer medulla. Similarly, the same group also identified unique features in macrophage subtypes relating to RNA kinetics and cell differentiation, which could not be identified by the traditional flow cytometry-based classification of M1/M2 phenotypes. Overall, combination of spatial and single cell RNA-seq methods and analytical approaches are powerful new tools that can provide new insight into the mechanisms of AKI pathogenesis and facilitate the development of potential new targets for treatment.

The study of proteinuria during CKD progression has been particularly challenging because the mechanisms underlying glomerular disease are many and include paracrine, inflammatory, immune, fibrotic, proliferative, metabolic, and apoptotic processes. Similarly, in the tubulointerstitial compartment multiple inflammatory and pro-fibrotic pathways contribute to the sustained fibrogenesis and renal damage progression. Historically, *in situ* hybridization methods have been applied for validation of bulk and/or scRNA seq data (48). The applications of ST, on the other hand, have the potential to advance the field; however, to date, no peer-reviewed data encompassing different etiologies of CKD and including both the glomerulus and tubules have been reported. Using ST, it will be possible to study different human glomerulopathies and key observations will presumably indicate the significance of interglomerular differences during injury that may prove to be essential for understanding how damage to the glomerulus is initiated and how these processes prime the kidney for the onset of proteinuria. Thus, the use of ST provides great potential to advance our understanding of CKD, and ultimately promote the development of personalized medicine approaches for the treatment of kidney diseases.

Different ST platforms have specific limitations; but they all share some drawbacks that include high processing costs, tissue processing requirements that often are challenging and laborious as well as region of interest resolution returning averaged transcriptional information from several cells within the specified area of interest (49), thus hindering the discovery of cell type-specific spatial patterns of localization and expression. In addition, formalin fixed paraffin embedded (FFPE) tissue preservation remains common practice, but most available ST methods only work satisfactorily on fresh frozen tissue, where morphological structure analysis is inferior to FFPE. Biological limitations in regard to glomerular mesangial cell identity crisis and molecular signature described for the scRNA-seq holds true also for ST but is mitigated by the possibility to spatially determine their localization. Lastly, a major drawback of all current techniques is that they only provide a snapshot in time of a dynamically active tissue (49). Therefore, samples derived at different time points would need to be studied independently to evaluate the spatiotemporal transcriptomic signatures (49, 50). Nonetheless, considering that ST is still in its infancy, future optimizations and development will provide us more precise and efficient approaches to study kidney disease.

## *In Vitro* Approaches

Despite the availability of many *in vitro* tools, 2D and tissue engineering based systems are yet functionally incomplete models of the filtration unit of the kidney, thus limiting studies aimed at understating mechanisms of proteinuria. Our chances of understanding glomerular mechanisms of injury have significantly improved thanks to the development of novel approaches such as kidney organoids or more recent microfluidic systems.

Kidney organoids are self-organized three-dimensional aggregations of cells that can be derived from embryonic stem cells as well as induced pluripotent stem cells (iPSCs). A wide range of protocols have been developed and perfectioned over the years to obtain kidney organoids that closely resemble the *in vivo* environment (51). Their potential to study the pathophysiology of the kidney has energized efforts for the development of more complex, better structured organoids that more closely mimic the *in vivo* development and maturation. They have quickly become a great platform to study podocyte damage (52, 53) and genetic defects, thanks to the use of Clustered Regularly Interspaced Short Palindromic Repeats (CRISPR)-Cas9 technology or primary mutated iPSC lines (54–57). Despite the great advances in the field, kidney organoids still present several challenges for the study of the glomerular filtration barrier and for functional studies including limited or partial cell maturation (58), underrepresentation of specific renal cells (59–61) as well as lack of a proper glomerular basement membrane (GBM) (53). In fact, even though that glomeruli organoids show maturing GBM matrisome, detectable amounts of type IV collagen α3 and α4 chains, which are essential for the formation of the mature basement membrane, was never clearly presented (53). Moreover, no clear deposition of collagen IVα3α4α5 trimer (the major constituent of the GBM), and proof that podocytes are fully mature, as the sole producer of this specific collagen chains were clearly shown by western blot. Scalability of kidney organoids for high-throughput studies was improved thanks to bioprinting approaches (62). Common to all organoids including kidney organoids is the lack of a proper vascularization which limits both their long-term viability as well as their usefulness to study organ functionality including permselectivity and tubular reabsorption. Different approaches have been employed to improve vascularization including the combination of organoids and microfluidic chips (63), as well as implantation of kidney organoids under the kidney capsule of mouse hosts (64). In conclusion, these novel systems hold great potential in promoting our understanding of renal development and pathophysiology, but their practical use is still elusive and further development is required to allow them to become a critical tool. In particular, the potential that kidney organoids could evolve to allow the *in vitro* study of proteinuria, or mechanisms of proteinuria remains highly limited.

While kidney organoids aim to comprehensively recreate all the renal compartments *in vitro*, the development of a kidney-on-a-chip system has focused on the *in vitro* generation of tubules and glomeruli. Successful development of PT structures has been confirmed by many groups (65, 66). However, while tubular proteinuria (inability of tubules to reabsorb proteins) could be an interesting target for mechanistic as well as therapeutic studies (67), kidney tubules-on-a-chip have not been used to investigate these phenomena, its mechanisms of action or potential drug treatments.

Establishment of glomeruli-on-a-chip has been for long time challenged by architectural and functional hurdles as well as by sourcing of cells. In fact, the generation of a glomerulus-on-a-chip was first reported in 2016, almost a decade later compared to the corresponding models for renal tubules (68). In a common fashion for glomerulus-on-a-chip systems, podocytes and endothelial cells were separated in the chip by a porous polycarbonate membrane coated with basement membrane extracts. Assessment of proteinuria was performed by applying mechanical force to the endothelial side, leading to cell damage, loss of junctions, and changes to the cell's cytoskeleton as well as leakage (69). Deleterious effects of mechanical stress on leakage were also studied by Musah et al. in their iPSC generated glomerulus-on-a-chip system (70). Mechanical strain, achieved by stretching the flexible PDMS (polydimethylsiloxane) membrane using vacuum, lead to a greater expression of nephrin in podocytes and promoted functionality, expressed as albumin retention (70). Moreover, albuminuria was also confirmed in an adriamycin mediated injury model, further supporting the microfluidic system as a platform for pathophysiological studies (70). At the same time, Wang at al. reported the effects of high glucose treatment on albumin leakage in another personalized glomerulus-on-a chip system accompanied by oxidative stress and podocyte damage (71). In 2019, our lab reported the development of a glomerulus-on-a-chip based on a new approach that allowed the generation of a filtration barrier devoid of artificial membranes separating human podocytes and glomerular endothelial cells, thus allowing for improved interaction between the two cell layers as well as the *de novo* generation of GBM (72). Permselectivity under normal culture conditions as well as albuminuria following a variety of insults including puromycin aminonucleoside damage and high glucose exposure was confirmed. Notably, use of podocytes carrying a collagen IV mutation (typical of Alport Syndrome) lead to loss of permeability. We also established a model of autoimmune disease by using sera from membranous nephropathy patients, confirming albuminuria that was proportional to the clinical levels of proteinuria and PLA2R antibody titer in the donors (72). More recently, Xie et al. developed a glomerulus-on-a-chip using a novel approach based on extruded topographic hollow fibers and successfully confirmed permselectivity of their platform (73). Taken together, the exciting progress in the field of kidney microfluidic systems, while still in its initial stages, holds the promise for new discoveries based on molecular mechanisms of injury in the filtration barrier as well as the possibility to apply those findings for a more personalized therapeutic approach. The existing microfluidic systems reconstruct separate segments of the nephron, such as the glomerulus or the tubular compartment only and are thus limited in regard to creating a complete functional nephron that faithfully replicates the mechanical forces at play in the glomerular tuft and in the tubular compartment. Additional limitations of these *in vitro* systems include the absence of other key cells like, for example, mesangial cells or macula densa cells. The *in vitro* systems also lack immune cells, such as resident macrophages, which presumably play important maintenance role in the glomerulus (74, 75).

## Conclusions and Future Directions

**Figure 1:**
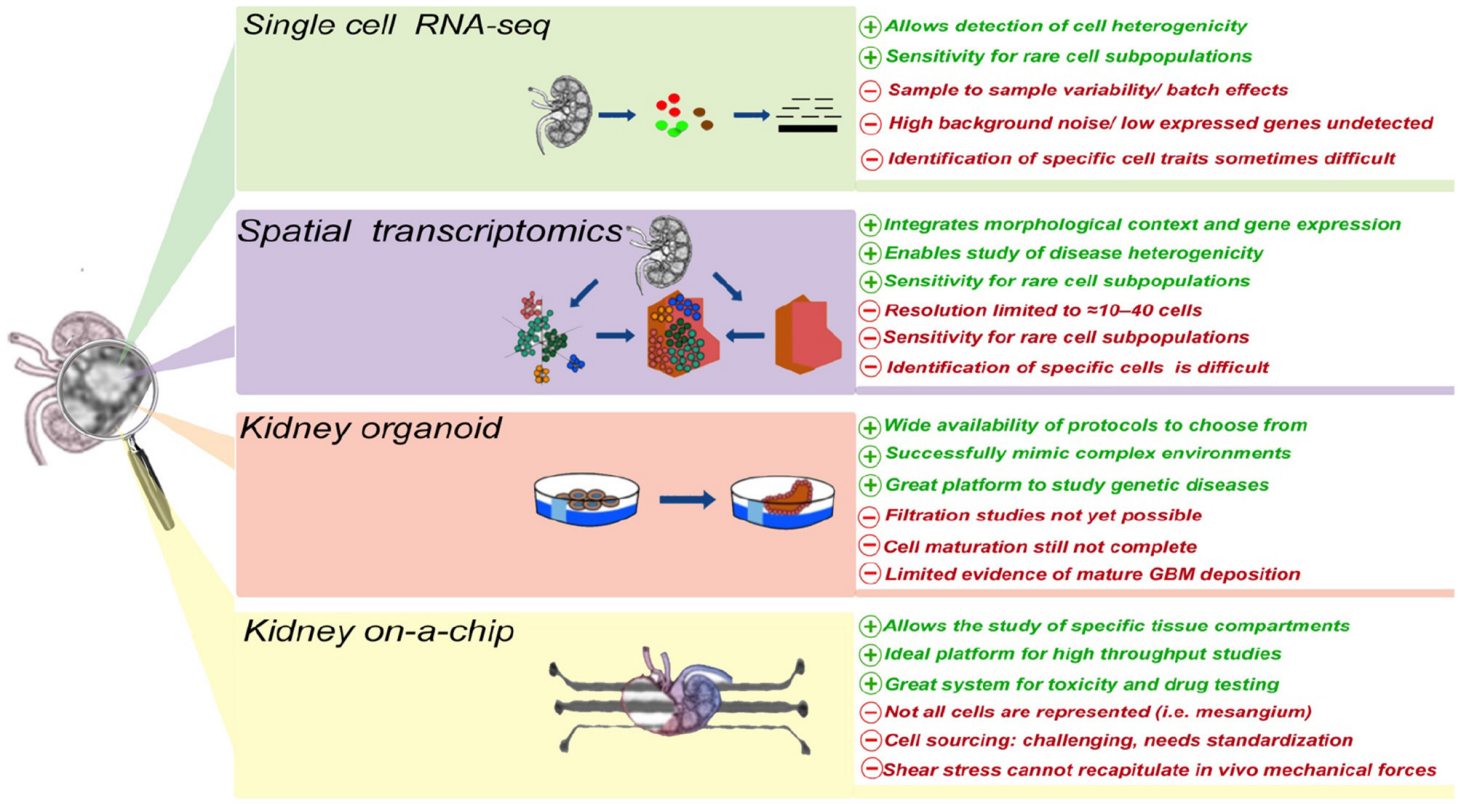
The advantages and disadvantages of different technologies currently applied to study cellular and molecular mechanism(s) of proteinuria.

In the last decade, the development of a wide array of research tools has significantly advanced our ability to better understand kidney disease, its origin, and its manifestations, including proteinuria. Single cell transcriptomics has the potential to enable the identification of the cell type (or types) initiating, promoting, or being damaged by proteinuria. Generation of these data is well complemented by the inclusion of spatial data. Additionally, development of kidney organoids and microfluidic systems has the potential to allow, for the first time, the *in vitro* study of proteinuric diseases, thus enabling both mechanistic and pre-clinical studies (Figure 1).

Despite the great promises, the use of these tools to study kidney disease and proteinuria is still in their infancy stage due to both cost and efficiency issues development of improved platforms, tailored and built upon the current needs in kidney research will bring, in the coming years, the much-needed tools to advance our knowledge of kidney disease.

## Author Contributions

EG, LP, SD, and SS conceptualized and wrote the manuscript. All authors contributed to the article and approved the submitted version.

## Conflict of Interest

The authors declare that the research was conducted in the absence of any commercial or financial relationships that could be construed as a potential conflict of interest.

## Publisher's Note

All claims expressed in this article are solely those of the authors and do not necessarily represent those of their affiliated organizations, or those of the publisher, the editors and the reviewers. Any product that may be evaluated in this article, or claim that may be made by its manufacturer, is not guaranteed or endorsed by the publisher.
